# Acknowledgment of Reviewers, 2023

**DOI:** 10.1200/GO.23.00246

**Published:** 2023-09-22

Peer review is at the core of scientific progress. It is through the careful critique of peer reviewers that manuscripts are scrutinized, evaluated, and improved. An article that has been peer reviewed is determined to be valid and worthy of publication. These peer-reviewed manuscripts are a critical component advancing our existing understanding of science.

As such, scientific outlets in general, and *JCO Global Oncology* in particular, owe an immense debt of gratitude to our volunteer peer reviewers. *JCO GO* strives to provide the highest quality peer review, so in recognition of Peer Review Week 2023 and its theme of Peer Review and The Future of Publishing, we are pleased to recognize your efforts and thank you for your expertise and time by presenting this list of those who have contributed to our success through peer review.

For your hard work in 2022-2023, our heartfelt thank you!

An asterisk indicates reviewers who have reviewed three or more manuscripts in 2022-2023. The dagger indicates reviewers who participated in the ASCO Reviewer Mentoring program. The double dagger indicates reviewers who participated in the ASCO Editorial Fellowship program. And the section sign indicates a colleague who assisted with reviews.

Ahmad Sufian Ab Rahman

Mohaned Abass

Innocent Abayo

Haneen Abaza*

Ahmed Abbasi

Maya Abdallah

Raafat Abdel Malek

Mohamed Abdelbaki

Abdelhafeez Abdelhafeez

John Tabiri Abebrese

Amitha Abraham

Aby Abraham

Afua Abrahams

Maria Graciela Abriata

Andres Acevedo

Bibek Acharya

Aleesha Adatia

Bolanle Adegboyega

Prisca Adejumo

Ademola Adeyeye

Abish Adhikari

Sonal Admane†

Jai Prakash Agarwal

Ajay Aggarwal

Alia Ahmad*

Larry Akoko

Murat Akova

Abd El Aziz Al Hammad

Raafat Alameddine

Tit Albreht

Thierry Alcindor

Saleh Alessy*

Firas Alniaimi

Abdulla Al-Rashdan*

Shaymaa AlWaheidi

Nada Alwan

Snegha Ananth

Benjamin Anderson

Ana Arance

Luca Arcaini

James Armitage

Ramandeep Arora

Surein Arulananda

Fredrick Asirwa

Burca Aydin

Roshani Babu

Warren Bacorro

Farhana Badar

Obsie Baissa

Joanmarie Balolong-Garcia

Deepak Bansal

Pedro Barata

Regina Barragán-Carrillo‡

Karen Bartholomew

Michael Barton

Philip Baum*†

Asim Belgaumi

Abubakar Bello

Javier David Benitez Fuentes

Cristiane Bergerot

Alice Bergin

Alejandro Berlin

Scott Berry

Naleen Raj Bhandari

Rohini Bhatia

Tiago Biachi de Castria

Eric Bing

Ahitagni Biswas

Stephanie Blank

Barri Blauvelt

Nancy Bolous

Josep Maria Borras

Eric Bouffet

Maria Bourlon*

Hasan Brau Figueroa

Lior Braunstein

Ari Brooks

Geoffrey Buckle

Alexandra Bukowski

Anna Cabanes

Santiago Cabezas-Camarero

Andrea Cappellano

Christine Carrington

Andrea Castro-Sanchez

Eduardo Cazap

Robert Chamberlain

Jason Yongsheng Chan

Guillermo Chantada

Abhishek Chatterjee

Yanin Chavarri Guerra

Mahati Chittem

Supriya Chopra*

Melvin Chua*

Linus Chuang

Hiu Shun Chung

Al Covens

Nicola Creagh

Alan Dal Pra

Amy Davies

Daphne Day

Geoff Delaney

Georgia Demetriou

Rosdali Yesenia Diaz Coronado

Abbey Diaz*

Colleen Dickie

Nazli Dizman

Darja Djordjevic

Soniya Dulal

Gadiel Dumlao

Henry Ebili

Verena Ehrbar

Marc Eid*†

Sebatian Ekenze

Khalid El Bairi

Alaa Elhaddad

Mahmoud El-Tamer

Ahmed Elzawawy

Jesper Eriksen

Mustafa Erman

Juliana Escobar

Manuel Espinoza Gutarra

Adebukola Ewuola

Muhammad Salman Faisal†

Oumaya Fawaz

Ethan Feinstein

Mario Fontes-Sousa

Tamer Fouad§

Marie Belle Francia

Julia Freckelton§

Soad Fuentes-Alabi

Takeo Fujii

Robert Peter Gale

Trivadi Ganesan

Vikas Garg

Jennifer Geel

Sushil Giri

Meredith Giuliani*

Vishnu Gopal

Lisa Grech

Rachel Greenup

Bhupesh Guleria

Ms Sabhyata Gupta*

Tejpal Gupta*

Marianne Guren

Jason Gurney

Bishal Gyawali

Sudip Haldar

Omar Hamidy§

Varsha Hande*

Mhamed Harif

Caleb Harris

Mona Hassan*‡

Anthony Herbert

Laila Hessissen

Tarek Hijal

Andreas Hinz

John Horan

Mary Isaacson

Amy Israel

Christopher Jackson

Paul Jacobsen

Karan Jatwani

Umesh Jayarajah

Jinani Jayasekera

Aarthi Jayraj*†

Barbara Jereczek-Fossa

Chandan Jha

Kunal Jobanputra

Deepa Joseph

Jin Hyoung Kang

Pashtoon Kasi

Jamal Khader

Yitbarek Mengistu Kibret

Saadettin Kilickap

Dennis Dong Hwan Kim

Stephen Kimani

Irem Koc

Anne Koch

Bogda Koczwara

Minjoung Koo

Kaladada Korubo

Rohini Kulkarni

Rajat Kumar

Anuj Kumar

Takayasu Kurata

Nilgun Kurucu

Jennifer Lai

Matteo Lambertini

Premila Leiphrakpam

Theodore Levin

Jeremy Lewin

Ya Huei Li

Tong Li

Yexiong Li

Bob Li*

Elizabeth Liow

Dorothy Lombe*

Mehraju Lone

Herbert Loong

Susana Lozano Esparza

Sandra Luna-Fineman

Humera Mahmood*

Pablo Mando

Belinda Mandrell

Diah Martina*

Leo Masamba

Aju Mathew

Jean-Marc Maurel*

Sam Mbulaiteye

Casey McAtee

James McCracken

Daniel Medina§

Santosh Menon

Rashmi Menon

Brandon Meyers

Vivienne Milch

Kenneth Miller

Michael Milosevic*

Prachi Mittal*

Laura Moliner†

Michalina Montano

Lesley Moody

Daniel Moreira

Jennifer Morgan

Deborah Mukherji

Melinda Mushonga§

Mohmmed Mutar

Alex Mutombo Baleka

Valerian Mwenda

Yoshiro Nakahara

Megha Nandwani

Long Nguyen*

Dietger Niederwieser

Monica Niger

Manjunath Nookala Krishnamurthy

Charles Normand

Maliha Nusrat

Andrew Odhiambo

Daniel O'Neil

Ahmad Ozair*†

Sandro Pasquali

Bishal Paudel

Shahana Pervin

Barbara Pistilli

Maya Prasad

Boby Putra§

Ryan Rader§

Meena Rafiq

Ajay Raghunath*

Vinod Ramani

Juan Carlos Ramos

Karthik Rao

Gregory Reaman

Lorna Renner

Josep-Maria Ribera

Rachel Riechelmann

Rawad Rihani

Carlos Rodriguez-Galindo

Paul Rogers

Eliane Rohner

Arya Mariam Roy

Diego Sadler

Solmaz Sahebjam

Edwin Saji§

Akash Sali

Marguerite Samson

Bruno Sangro

Ursula Sansom-Daly

Matthew Schlumbrecht

Aba Scott*

Jason Semprini

Jan Seuntjens

Jaime Shalkow

Omar Shamieh

Omshree Shetty

Ramila Shilpakar*

Akihiko Shimomura

Lawrence Shulman

David Shultz

Iman Sidhom

Hannah Simonds

Randeep Singh

Shwetabh Sinha

Bhawna Sirohi

Jeremy Slone

Montserrat Soler*

Enrique Soto§

Luis Souhami

Dan Stark

Martin Stockler

Anand Swaminath

Farzad Taghizadeh-Hesary

Charles Jeffrey Tan

Sean Tan*

Laure-Anne Teuwen*

Yu Tian

Anil Tibdewal

Nataly Torrejon†

Edward Trimble

Derek Tsang

Craig Underhill

Karla Unger-Saldana

Verna Vanderpuye*

Rebecca Venchiarutti*

Shalini Vinod

Yang Wang§

Don Thiwanka Wijeratne*

Austin D. Williams

Brooke Wilson

Jeff Winter

Rebecca Wong

Budhi Yadav*

Sanjay Yadav

Bilgehan Yalcin

Mei Ling Yap*

Fabio Ynoe de Moraes*

Muhammad Aasim Yusuf

Emily Zabor

Roberto Zanetti

Veronica Zavagli

Nik Zeps*

Jian-Guo Zhou

Hongcheng Zhu*

Eduardo Zubizarreta

